# Epidemiology of hospitalised paediatric community-acquired pneumonia and bacterial pneumonia following the introduction of 13-valent pneumococcal conjugate vaccine in the national immunisation programme in Japan

**DOI:** 10.1017/S0950268820000813

**Published:** 2020-04-17

**Authors:** N. Takeuchi, S. Naito, M. Ohkusu, K. Abe, K. Shizuno, Y. Takahashi, Y. Omata, T. Nakazawa, K. Takeshita, H. Hishiki, T. Hoshino, Y. Sato, N. Ishiwada

**Affiliations:** 1 Department of Infectious Diseases, Medical Mycology Research Centre, Chiba University, Chiba, Japan; 2 Department of Paediatrics, Chiba University Hospital, Chiba, Japan; 3 Department of Paediatrics, Chiba Kaihin Municipal Hospital, Chiba, Japan; 4 Department of Clinical Laboratory, Chiba Kaihin Municipal Hospital, Chiba, Japan; 5 Department of Paediatrics, Seikeikai Chiba Medical Centre, Chiba, Japan; 6 Division of Infectious Diseases, Chiba Children's Hospital, Chiba, Japan; 7 Department of Preventive Medicine and Public Health, Keio University School of Medicine, Shinjuku-ku, Japan

**Keywords:** Paediatrics, pneumococcal infection, pneumonia, vaccination (immunisation)

## Abstract

Studies on community-acquired pneumonia (CAP) and pneumococcal pneumonia (PP) related to the 13-valent pneumococcal conjugate vaccine (PCV13) introduction in Asia are scarce. This study aimed to investigate the epidemiological and microbiological determinants of hospitalised CAP and PP after PCV13 was introduced in Japan. This observational hospital-based surveillance study included children aged ⩽15 years, admitted to hospitals in and around Chiba City, Japan. Participants had bacterial pneumonia based on a positive blood or sputum culture for bacterial pathogens. Serotype and antibiotic-susceptibility testing of *Streptococcus pneumoniae* and *Haemophilus influenzae* isolates from patients with bacterial pneumonia were assessed. The CAP hospitalisation rate per 1000 child-years was 17.7, 14.3 and 9.7 in children aged <5 years and 1.18, 2.64 and 0.69 in children aged 5–15 years in 2008, 2012 and 2018, respectively. There was a 45% and 41% reduction in CAP hospitalisation rates, between the pre-PCV7 and PCV13 periods, respectively. Significant reductions occurred in the proportion of CAP due to PP and PCV13 serotypes. Conversely, no change occurred in the proportion of CAP caused by *H. influenzae*. The incidence of hospitalised CAP in children aged ⩽15 years was significantly reduced after the introduction of PCV13 in Japan. Continuous surveillance is necessary to detect emerging PP serotypes.

## Introduction

*Streptococcus pneumoniae* is one of the leading causes of community-acquired pneumonia (CAP) in children. After the introduction of heptavalent pneumococcal conjugate vaccine (PCV7), the incidence of paediatric CAP decreased in many countries [1–3]. The 13-valent pneumococcal conjugate vaccine (PCV13) against paediatric CAP, as reported recently, is effective in several countries [4–7]. In the USA, PCV7 and PCV13 were introduced in 2000 and 2010, respectively. Griffin *et al*. [8] reported that all-cause pneumonia hospitalisations in children aged <2 years declined by an additional 27%, between 2010 and 2012 in the USA following the introduction of PCV13. The reduction in all-cause pneumonia admissions in children provides an estimate of the proportion of paediatric pneumococcal pneumonia (PP); however, little is known about the incidence of pneumonia attributable to *S. pneumoniae* and individual serotypes.

According to the review on paediatric pneumonia hospitalisation incidence rates in developed countries, the incidence of pneumonia hospitalisations in Japan was higher than that of other developed countries before the introduction of PCV [9]. PCV7 was introduced in Japan in February 2010 as a voluntary vaccination. One year later, the Japanese government subsidised the vaccination. Most of the children <5 years of age were able to receive the PCV7 immunisation free of charge. The estimated immunisation coverage increased rapidly to >95% and high immunisation coverage was maintained. PCV7, included in the routine vaccination programme in April 2013 was changed to PCV13 in November 2013 [10]. The schedule for PCV13 vaccination in Japan is three doses, administered at the ages of 2, 3 and 4 months, followed by a booster dose at 12–15 months. Both the subsidised vaccination and routine vaccination have the same schedule in Japan. However, compensation for vaccine-related health damage differs.

To assess the direct impact of PCV against paediatric CAP, we implemented a surveillance system before the introduction of PCV7 in Chiba City, Japan. Based on this surveillance system, we determined the hospitalised paediatric CAP incidence for children <5 years of age, which showed that PP decreased significantly [11, 12].

Here we report the incidence of hospitalised CAP after the introduction of PCV13 in the national immunisation programme in Japan, based on this surveillance system [11, 12]. We also described the serotype, sequence type (ST) and antimicrobial susceptibility of *S. pneumoniae* isolated from patients with PP after the introduction of PCV13. To date, detailed reports on the aetiology of CAP related to the PCV13 era in Asian and Western Pacific countries are scarce. Such information, which would enable an estimate of the impact of PCV13 against CAP in children, is essential in these countries where pneumonia is the leading cause of death in children [13].

## Methods

### Study design, study setting and participants

The incidence of hospitalised CAP was calculated based on an observational surveillance study in Chiba City, the capital of Chiba Prefecture, in central Japan. According to the Japanese census data, the total population of Chiba City was 969 544 (about 0.8% of the population of Japan) [14] and the population of children aged <5 years and 5–15 years in Chiba City was 35 885 and 92 281, respectively, in September 2018 [14]. A questionnaire was sent to 15 hospitals with paediatric wards in and around Chiba City. The study occurred from April 2016 to March 2019 (Japanese fiscal year 2016–2018). Children aged 1 month to 15 years who lived in Chiba City and were admitted to hospital with CAP were included in this study. The number of hospitalised CAP cases covered the entire Chiba City and the entire region, including residents only.

The 15 hospitals are located in and around Chiba City. There is little possibility of admitting paediatric CAP patients who are resident in Chiba City, other than in the 15 hospitals. The numbers of hospital admissions due to pneumonia and blood culture-positive pneumonia patients were estimated to cover all inhabitants of Chiba City aged ⩽15 years whose data were obtained from all hospital clinical records.

### The incidence of hospitalised CAP in Chiba City

The incidence of hospitalised CAP in this study period was compared with that reported in our previous studies from April 2008 to March 2009 (2008) and from April 2012 to March 2013 (2012) [11, 12]. The number of hospitals targeted in this surveillance was lower than that in our previous studies [11, 12]. In Japan, some hospitals closed the paediatric wards as the number of children decreased. In this study, we included all seven hospitals with a paediatric ward in Chiba City and eight nearby hospitals with paediatric wards. Therefore, the 15 hospitals included in this study covered almost all hospitalised children who were residents in Chiba City. To calculate the annual hospitalised CAP incidence in Chiba City for those < 5 years of age and those aged 5–15 years in 2016–18, the number of hospitalised CAP cases was divided by the total number of inhabitants pertaining to the two age groups (obtained from Japanese census data). Person-years were based on mid-year population estimates. There are six health care centres in Chiba City. Health care centres only care for healthy children in Japanese health system. In general, radiological diagnosis of pneumonia is done by a paediatrician not by radiologists, in Japan. In this study, abnormal shadows in chest radiographs were confirmed by at least two paediatricians. Pneumonia was diagnosed based on at least one of the following abnormal clinical findings on chest radiograph: fever, cough, rapid breathing, difficulty in breathing, or crackles on auscultation of the lungs. The same doctors in each hospital read the chest radiographs and made the diagnosis of pneumonia. In each hospital, the member of senior medical staff did not change compared to our previous studies. Furthermore, the accuracy of the radiological diagnosis of pneumonia in this study remained similar to that of our previous studies.

### Diagnosis of bacterial pneumonia

Blood cultures and sputum samples were collected from the patients on admission. Bacterial pneumonia was diagnosed based on a positive blood culture or the isolation of microorganisms from washed sputum samples. Sputum samples were collected from children as described previously [15]. Collected sputum samples were washed with sterilised saline solution. A small purulent portion of the washed sputum was smeared onto glass slides. Valid Gram-stained smears for sputum culture samples were according to Geckler's classification four or five. This method effectively isolated the pathogenic bacteria (e.g. *S. pneumoniae*, *H. influenzae* and *Moraxella catarrhalis*) and reduced contamination by oral flora. Washed sputum samples were cultured in each hospital and pathogens accounting for >50% of the colonies in the culture, or presenting as >1 × 10^7^ cfu/ml were regarded as pathogenic. In order to diagnose *M. catarrhalis* pneumonia, leukocyte phagocytosis of bacteria was detected in Gram-stained samples [16].

Data on patients' backgrounds and clinical information were collected and recorded on a standard case report sheet. Administration of PCV7 and/or PCV13 was documented in the patient's medical record or in the maternal health record book that is used to document children's vaccination history in Japan. Vaccination status information was obtained from all patients in four hospitals. The number of hospitalised bacterial pneumonia in children aged ⩽15 years who were admitted to hospital with pneumonia was determined during the three vaccination periods in four hospitals in Chiba City. These four hospitals reported more than half of the hospitalised pneumonia patients in our previous studies [11, 12].

The number of hospitals where bacterial analyses were performed were 6, 5 and 4 in 2008, 2012, 2016–18, respectively. Except in two hospitals, the number of the paediatricians decreased and doctors predominantly took care of outpatients during the study period. Therefore, we thought that the coverage of hospitalised CAP patients was similar in the three study periods.

### Isolation of *S. pneumonia* strains

*S. pneumoniae* isolates from blood and washed sputum samples were collected from the four study hospitals. The strains were stored at −80 °C in each hospital and sent to the Medical Mycology Research Centre at Chiba University. This centre used the same methods and had the same skill of bacteriological analysis compared with that in our previous studies. Each isolate was grown on trypticase soy agar (TSA) with 5% sheep blood (Nippon Becton Dickinson Co. Ltd., Tokyo, Japan) for 24 h at 37 °C in 5% CO_2_. Each isolate was identified as *S. pneumoniae* using an optochin susceptibility test and a bile solubility test. Polymerase chain reaction (PCR) assays targeting the *lytA* gene, which encodes the major pneumococcal autolysin *(LytA*), were also used to identify *S. pneumoniae*. All strains were susceptible to optochin, bile-soluble and positive for the *lytA* gene and were therefore identified as *S. pneumoniae*.

Serotypes were determined using the slide agglutination reaction with the *S. pneumoniae* antisera using the Seiken set (Denka Seiken, Tokyo, Japan) and the Quellung reaction using pneumococcal antisera (Statens Serum Institut, Copenhagen, Denmark). The non-encapsulated strain was detected via the PCR method using primers for *cpsA*, the capsular polysaccharide biosynthesis gene.

Multilocus sequence typing (MLST) was performed as described previously [17]. Sequence types (STs) were determined by comparing the derived sequences of each locus to all known alleles by reference to the MLST database (http://pubmlst.org/spneumoniae/). The STs were compared with 43 pneumococcal clones, which included 26 multidrug-resistant (MDR) clones, in the Pneumococcal Molecular Epidemiology Network (PMEN; http://www.sph. emory.edu/PMEN/). Strains were assigned to one clonal complex (CC) when five or six of the seven alleles were identical to those of another ST in the group of the relationships between the isolates were determined using goeBURST software, Version 1.2.1 (http://www.phyloviz.net/goeburst).

Antimicrobial susceptibilities of *S. pneumoniae* to penicillin G (PCG), ampicillin (ABPC), cefditoren (CDTR), cefotaxime (CTX), ceftriaxone (CTRX), meropenem (MEPM), panipenem (PAPM), tebipenem (TBPM), erythromycin (EM), clindamycin (CLDM), tosufloxacin (TFLX) and vancomycin (VCM) were analysed using the broth microdilution method according to the Clinical and Laboratory Standards Institute (CLSI) M07-A11 protocol. The minimal inhibitory concentration (MIC) breakpoints were defined according to the CLSI criteria (CLSI M100-S29).

### Isolation of *H. influenzae* strains

*H. influenzae* isolates from blood and sputum samples were stored at −80 °C in each hospital and sent to the Medical Mycology Research Centre at Chiba University for further testing. The strains were stored immediately after isolation and then grown on Chocolate II Agar (Nippon Becton Dickinson Company, Tokyo, Japan). Strains were identified based on their growth requirements for hemin and nicotinamide adenine dinucleotide (X and V factors). They were also identified genetically by the PCR method as described previously, using the primers for sialic acid transporter gene (*siaT*) of *H. influenzae* [18].

Serotyping was confirmed by PCR using primers for *bexA* and serotype-specific genes, as described previously [19]. Antimicrobial susceptibilities of *H. influenzae* to PCG, ABPC, CDTR, CTX, CTRX, MEPM, PAPM, TBPM, EM, CLDM, TFLX and VCM were analysed using the broth microdilution method according to the CLSI M07-A11 protocol. MIC breakpoints were defined according to the CLSI criteria (CLSI M100-S29). *β*-lactamase production was tested by cefinase disk (Nippon Becton Dickinson Company, Tokyo, Japan).

### Statistical analysis

All data were analysed using JMP Pro Version 12 (SAS Institute, Cary, NC). Poisson regression was used to estimate incidence rates, incidence rate ratios and confidence intervals of CAP and PP. Between-group differences in patient characteristics were analysed using the Kruskal–Wallis test. Fisher's exact test was used to compare the coverage rate of PCV13 serotypes of *S. pneumoniae* isolated from PP patients before and after the introduction of PCV7/PCV13. All odds ratios, 95% confidence intervals (CIs) and *P*-values were estimated using logistic regression based on Firth's penalised likelihood estimation. All *P*-values represented two-tailed tests, with *P* < 0.05 considered statistically significant.

### Ethical issues

The authors assert that all procedures contributing to this work complied with the ethical standards of the relevant national and institutional (Chiba University Ethics Committee [No. 1301]) committees on human experimentation and with the Helsinki Declaration of 1975, as revised in 2008. Patients' records and information were anonymised prior to the analysis. For the microbial study performed in four hospitals, written informed assent was obtained from the parents of the children with CAP at the time of admission, in accordance with the guidelines of the Institutional Review Board of Chiba University (Chiba University Ethics Committee [No. 1301]).

## Results

### The annual hospitalised CAP incidence among children aged <5 years

**Fig. 1.**
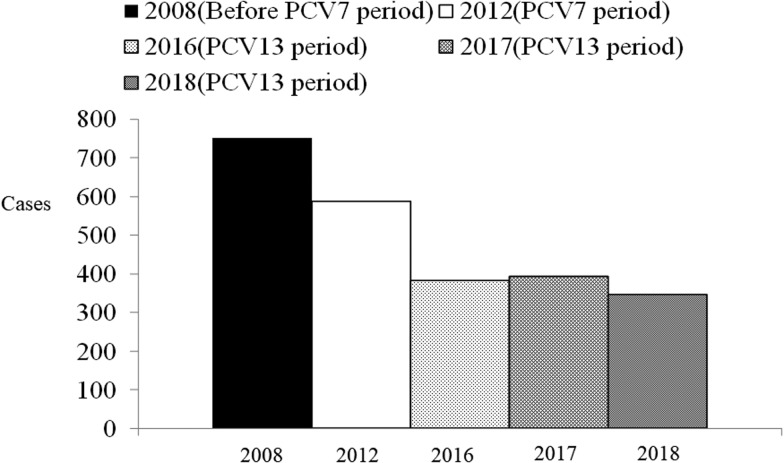
Number of hospitalized paediatric children <5 years of age with CAP before and after PCV7/PCV13 introduction in Chiba City, Japan. Since the introduction of PCV in children, the incidence of hospitalised paediatric CAP in children aged <5 years has decreased. After PCV13 introduction, the incidence of hospitalised CAP children <5 years of age further declined. CAP, community-acquired pneumonia; PCV7, heptavalent pneumococcal conjugate vaccine; PCV13, 13-valent pneumococcal conjugate vaccine

Overall, 1399 children were admitted with CAP, in the PCV13 period. Of the children who participated in the study, 596 (42.6%), 526 (37.6%) and 277 (19.8%) were aged <2, 2–4 and 5–15 years, respectively. Figure 1 shows the number of children in Chiba City hospitalised with CAP in the pre-PCV7, PCV7 and PCV13 periods. The annual hospitalised CAP incidence per 1000 children aged <5 years in 2008, 2012, 2016, 2017 and 2018 were 17.7, 14.3, 10.1, 10.6 and 9.7, respectively. The CAP hospitalisation rate in children <5 years of age in 2018 declined by 45% (incidence rate ratio (IRR) 0.55, 95% confidence interval (CI) 0.48–0.62) and 32% (IRR 0.68, 95% CI 0.59–0.77) compared to the rates in the pre-PCV7 and PCV7 periods, respectively (Table 1).

**Table 1.** The incidence of hospitalized community-acquired pneumonia in children in Chiba city, Japan, before and after the introduction of PCV7 and PCV13

	2008 (Before PCV7 period)	2012 (PCV7 Period)	2018 (PCV13 Period)	2018 *vs*. 2008^b^		2018 *vs*. 2012^b^	
	Incidence rate^a^ (No. of cases)	Incidence rate^a^ (No. of cases)	Incidence rate^a^ (No. of cases)	IRR (95% CI)	*P* value	IRR (95% CI)	*P* value
CAP <5 years	17.7 (752)	14.3 (588)	9.7 (347)	0.55 (0.48–0.62)	<0.001	0.68 (0.59–0.77)	<0.001
CAP 5–15 years	1.18 (115)	2.64 (259)	0.69 (64)	0.59 (0.43–0.80)	<0.001	0.26 (0.20–0.35)	<0.001

CAP, community-acquired pneumonia; CI, confidence interval; IRR, incidence rate ratio; PCV7, heptavalent pneumococcal conjugate vaccine; PCV13, 13-valent pneumococcal conjugate vaccine.

a Cases/1000 population per year.

b Estimated using Poisson regression.

### The annual hospitalised CAP incidence among children aged 5–15 years

**Fig. 2.**
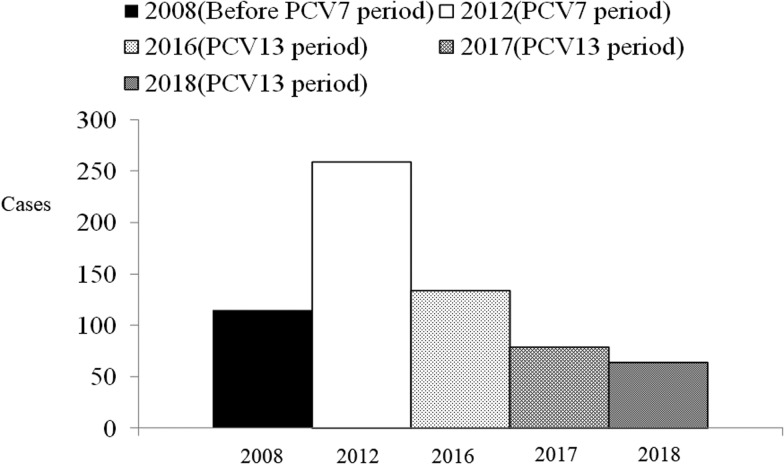
The number of hospitalised paediatric children 5–15 years of age with CAP before and after PCV7/PCV13 introduction in Chiba City, Japan. After PCV13 introduction, the incidence of hospitalised CAP children 5–15 years of age declined. CAP, community-acquired pneumonia; PCV7, heptavalent pneumococcal conjugate vaccine; PCV13, 13-valent pneumococcal conjugate vaccine

The annual hospitalised CAP incidence per 1000 children aged 5–15 years in 2008, 2012, 2016, 2017 and 2018 were 1.18, 2.64, 1.41, 0.84 and 0.69, respectively (Fig. 2). The CAP hospitalisation rate in children aged 5–15 years increased in the PCV7 period compared to the pre-PCV7 period. However, in the PCV13 period, the rate declined by 41% (IRR 0.59, 95% CI 0.43–0.80) and 74% (IRR 0.26, 95% CI 0.20–0.35), compared to the rates in the periods before and after PCV7, respectively (Table 1).

### Characteristics of children admitted to the four study hospitals with CAP

Overall, 904 of 1399 (64.6%) children were admitted to four study hospitals with the diagnosis of CAP in the PCV13 period. During the study period, seven children with bacteremic pneumonia caused by *S. pneumoniae* were reported. Their characteristics are shown in Table 2. One child died of CAP due to parainfluenza virus.

**Table 2.** The characteristics of children with community-acquired pneumonia admitted to the four study hospitals after the introduction of PCV13

	Total (*N* = 904)	2016 (*N* = 315) *n* (%)	2017 (*N* = 309) *n* (%)	2018 (*N* = 280) *n* (%)	*P* value
Age (years)					
0–1	430	134 (42.5)	164 (53.1)	132(47.1)	0.02
2–4	313	109 (34.6)	104 (33.7)	100 (35.7)	
5–15	161	72 (22.9)	41 (13.3)	48 (17.1)	
Sex					
Male	470	180 (57.1)	152 (49.2)	138 (49.3)	0.14
Female	434	135 (42.9)	157 (50.8)	142 (50.7)	
Underlying disease					
Present	351	121 (38.4)	125 (40.5)	105 (37.5)	0.34
Absent	553	194 (61.6)	184 (59.5)	175 (62.5)	
Antibiotic prescription before admission					
Yes	346	139 (44.3)	109 (35.3)	98 (35.0)	<0.001
No	558	176 (55.7)	200 (64.7)	182 (65.0)	
History of PCV					
PCV13, 4 shots	415	100 (31.7)	151 (48.9)	164 (58.6)	<0.001
PCV13, 1–3 shots	244	86 (27.3)	87 (28.2)	71 (25.4)	
PCV7 only, 1–4 shots	114	70 (22.2)	25 (8.1)	19 (6.8)	
None	59	23 (7.3)	22 (7.1)	14 (5.0)	
Unknown	72	36 (11.4)	24 (7.8)	12 (4.3)	
History of Hib vaccine					
4 shots	530	160 (50.8)	184 (59.5)	186 (66.4)	0.002
1–3 shots	243	94 (29.8)	80 (25.9)	69 (24.6)	
None	59	25 (7.9)	21 (6.8)	13 (4.6)	
Unknown	72	36 (11.4)	24 (7.8)	12 (4.3)	

PCV13, 13-valent pneumococcal conjugate vaccine; PCV7, heptavalent pneumococcal conjugate vaccine; Hib, *Haemophilus influenzae* type b

### Pneumococcal pneumonia

We obtained sputum and blood samples from 828 (91.6%) and 819 (90.6%) of the 904 children with CAP. While *S. pneumoniae* was culture-dominant in 64/828 (7.7%) of the sputum samples, there were only six blood culture samples positive for *S. pneumoniae*. Six patients had blood culture samples positive for *S. pneumoniae*, four (4/276: 1.5%) in 2016, one (1/ 292: 0.3%) in 2017 and one (1/ 251: 0.4%) in 2018. Of the six patients with blood culture-positive *S. pneumoniae*, three also had a positive sputum culture. One patient with positive blood culture in 2017, for *S. pneumoniae*. However, the patient was hospitalised to the remaining 11 hospitals included in our CAP study, which enabled us to obtain the pneumococcal strain for further analysis. *H. influenzae* was culture-dominant in 132/828 (15.9%) sputum samples, but no child was blood culture-positive for *H. influenzae*. Table 3 shows the microorganisms isolated from blood and sputum samples among CAP patients in the pre-PCV7, PCV7 and PCV13 periods. Over the course of the three periods, the number of isolates of *S. pneumoniae* decreased. In contrast to the incidence trend of CAP due to *S. pneumoniae* that related to *H. influenzae* and *M. catarrhalis* did not change significantly after PCV7 and PCV13 introduction.

**Table 3.** Microorganisms isolated from blood and dominantly isolated from sputum samples of children with community-acquired pneumonia admitted to the four study hospitals

	Before PCV7 period	PCV7 period	PCV13 period		
	2008 (*N* = 458)	2012 (*N* = 495)	2016 (*N* = 315)	2017 (*N* = 309)	2018 (*N* = 280)
*Streptococcus pneumoniae* no. (%)	75 (16.4)	41 (8.3)	19 (6.0)	24 (7.8)	24 (8.6)
Blood culture isolates	5	4	4	1	1
Sputum culture isolates	70	37	15	23	23
*Haemophilus influenzae* no. (%)	48 (10.5)	56 (11.3)	34 (10.8)	55 (17.8)	43 (15.4)
*Moraxella catarrhalis* no. (%)	13 (2.6)	16 (3.2)	12 (3.8)	13 (4.2)	22 (7.9)

PCV7, heptavalent pneumococcal conjugate vaccine; PCV13, 13-valent pneumococcal conjugate vaccine.

### Serotype distribution of *S. pneumoniae* strains

**Fig. 3.**
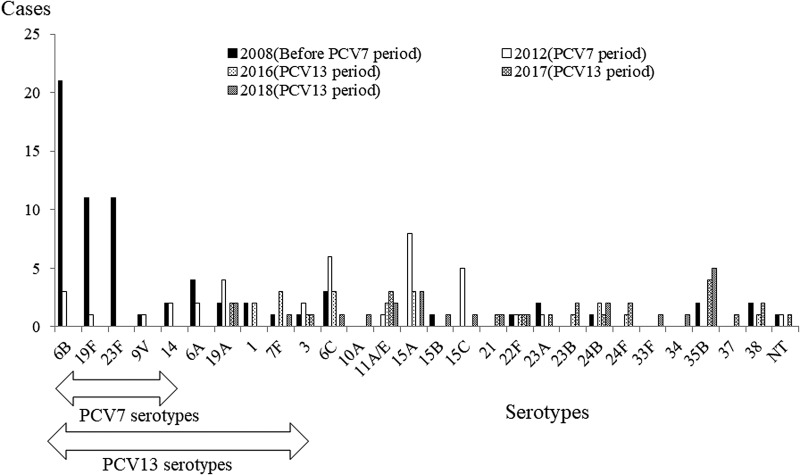
Distribution of *S. pneumoniae* serotype isolated from blood and sputum of the paediatric inpatients with CAP. In the pre-PCV7 period, the most frequent serotypes were 6B, 23F and 19F in children. After introduction of PCV7, PCV7 serotypes dramatically decreased and the major serotypes changed to 19A, 6C, 15A and 15C. In the PCV13 period, of the PCV13 serotypes, a small number of serotypes 1, 3, 7F and 19A were isolated. Of the non-PCV13 serotypes, the dominant serotypes were 35B, 15A and 11A/E.

Table 4 and Figure 3 show serotype distribution of *S. pneumoniae* strains isolated from blood and dominant isolates from sputum samples. Of the three children with *S. pneumoniae* isolated in both blood and sputum cultures, the blood isolates and sputum isolates were of the same serotype in two patients. In the third patient, the blood isolate was serotype 24B, while the sputum isolate was 24B capsular loss strain. In the pre-PCV7 period, the most frequent serotypes were 6B, 23F and 19F. Of the isolated strains, 67.6% and 82.4% were covered by PCV7 and PCV13, respectively. After PCV7 introduction, PCV7 serotypes dramatically decreased and the major serotypes changed to 19A, 6C, 15A and 15C. In the PCV13 period, among PCV13 serotypes, the incidence of CAP due to serotypes 1, 3, 7F and 19A strains decreased.

**Table 4.** PCV7/PCV13 serotypes of *Streptococcus pneumoniae* isolated from the blood or sputum as a proportion of all *Streptococcus pneumoniae* isolates in children with community-acquired pneumonia admitted to the four study hospitals, according to the year of admission

	Before PCV7 period	PCV7 period	PCV13 period		
	2008	2012	2016	2017	2018
PCV7 serotypes^a^	67.6% (46/68)	18.4% (7/38)	0.0% (0/20)	0.0% (0/22)	0.0% (0/23)
PCV13 serotypes^b^	82.4% (56/68)	39.5% (15/38)	30.0% (6/20)	13.6% (3/22)	13.0% (3/23)

PCV7, heptavalent pneumococcal conjugate vaccine; PCV13, 13-valent pneumococcal conjugate vaccine.

a *S. pneumoniae* serotypes targeted by the PCV7.

b *S. pneumoniae* serotypes targeted by the PCV13.

One strain was isolated from the blood in a child that was admitted to a hospital, other than the four study hospitals.

Among the non-PCV13 serotypes, the dominant serotypes were 35B, 15A and 11A after PCV13 introduction. The PCV13 serotype rates among all isolated strains gradually decreased from 30.0% to 13.0% after the introduction of PCV13. Among PCV13 serotypes, 75% of the strains were isolated from patients with underlying disease.

MLST was performed on 59 sputum isolates in which *S. pneumoniae* was the dominant organism on the six blood isolates in the PCV13 period (Table 5). Of the 65 isolates tested, 35 STs were found, including two new STs. STs 22F (ST1092), 23A (ST3163), 24B (ST2572 and ST162), 24F (ST2572 and ST5496), 34 (ST7388) and 38 (ST1429) were categorised into the same clonal group with ST320 by eBURST. Among tested strains, 23.7% (14/59) and 33.3% (2/6) of the sputum and blood isolates had STs identical to five international PMEN clones, respectively. Nine, seven and six strains of 35B-ST558, 11A-ST99 and 15A-ST63, respectively, were the three major ST types in the PCV13 period.

**Table 5.** Multilocus sequence typing analysis of *Streptococcus pneumoniae* strains isolated after the introduction of PCV13, by year

Vaccine type	Sero type	ST	CC	PMEN clones	Number of the strains			
					2016	2017	2018	Total
PCV13	1	306	306	Sweden^1^-28 (ST306)	2(1)			2
	3	180	180	Netherlands^3^-31 (ST180)	1	1		2
	7F	191	191	Netherlands^7F^-39 (ST191)	3(1)		1	4
	19A	2331	2331			1	1	2
		3111	3111			1	1	1
		6111	347					1
Non-PCV13	6C	2924	2924		1		1	2
		2974	439		1			1
		6183	2924		1			1
	10A	5236	113				1	1
	11A	99	99		2	3	2	7
	15A	63	63	**Sweden ^15A^-25 (ST63)**	3		3	6
	15B	199	199	Netherlands^15B^-37 (ST199)			1	1
	15C	199	199	Netherlands15B-37 (ST199)			1	1
	21	1233	1381			1		1
		14 709	1381				1	1
	22F	10 926	433		1			1
		433	433				1	1
		7314	433			1		1
	23A	3163	338			1		1
	23B	3746	Singleton		1			1
		546	546			1		1
		727	439			1		1
	24B	2572	2572		2(1)^a^	1(1)		3
		162	162				1	1
		2754	2754				1	1
	24F	2572	2572		1(1)	1		2
		5496	2572			1		1
	33F	717	717				1(1)	1
	34	7388	7388				1	1
	35B	558	558			4	5	9
	37	447	447			1		1
	38	6429	6429		1	1		2
		1429	320			1		1
	NT	14 708	230			1		1
Total					20	22	23	65

PCV7, heptavalent pneumococcal conjugate vaccine; PCV13, 13-valent pneumococcal conjugate vaccine; ST, sequence type; CC, clonal complex; UT, untypeable; NT, non-typeable; PMEN, Pneumococcal Molecular Epidemiology Network

NT strain was the non-encapsulated strain

Clones known as multidrug-resistant pneumococcal molecular epidemiology network (PMEN) clones are shown in bold.

The number of strains isolated from blood is shown in (parentheses).

a One strain that was isolated from the sputum was 24B capsular loss strain.

### Antimicrobial susceptibility of *S. pneumoniae*

**Fig. 4.**
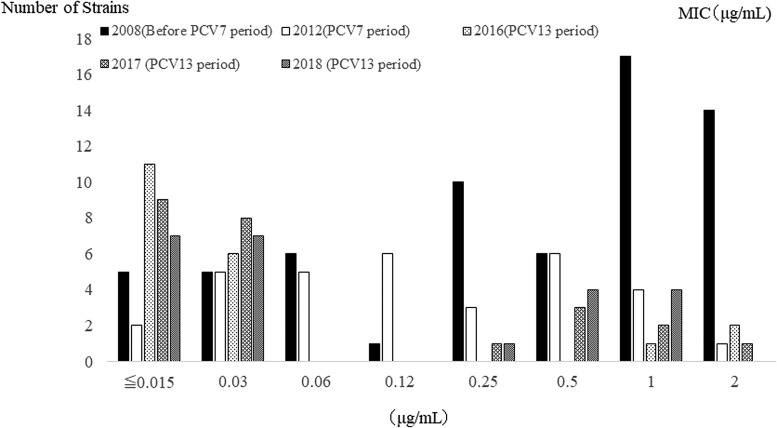
Susceptibility of *S. pneumoniae* isolates from blood and sputum samples to penicillin G among CAP patients. The susceptibility to PCG recovered after PCV introduction. CAP, community-acquired pneumonia; PCG, penicillin G; PCV, pneumococcal conjugate vaccine

Antimicrobial susceptibility was tested in 68 *S. pneumoniae* isolates. Table 6 shows MIC 50 and MIC-range of the isolates tested according to the year. Antimicrobial susceptibility did not change after the introduction of PCV13. Figure 4 shows the PCG susceptibility of *S. pneumoniae* isolates from blood and sputum samples among CAP patients during the pre-PCV7, PCV7 and PCV13 periods. The proportion of PCG-sensitive isolates increased after PCV introduction. In the PCV13 period, the serotypes that were less susceptible to PCG were serotypes 15A (ST 63) and 35B (ST 558). These serotypes were susceptible to the antibiotics, TFLX and TBPM, which were newly approved in Japan. From 2017, EM susceptible strains were detected. Of the total, 53% of PCG less-susceptible strains (PCG ⩾ 0.12) were isolated from patients with underlying disease.

**Table 6.** Antimicrobial susceptibility of *Streptococcus pneumoniae* isolates from children with community-acquired pneumonia

*S. pneumoniae*	2016		2017		2018	
Number of strains	20		25		23	
	MIC_50_	MIC range	MIC_50_	MIC range	MIC_50_	MIC-range
PCG	⩽0.015	⩽0.015 – 2	0.03	⩽0.015 – 2	0.03	⩽0.015 – 1
ABPC	⩽0.03	⩽0.03 – 4	0.06	⩽0.03 – 4	0.06	⩽0.03 – 4
CTX	⩽0.03	⩽0.03 – 0.5	0.25	⩽0.03 – 0.5	0.25	⩽0.03 – 0.5
CDTR	⩽0.03	⩽0.03 – 0.5	0.25	⩽0.03 – 0.5	0.25	⩽0.03 – 0.25
CTRX	⩽0.03	⩽0.03 – 0.5	0.12	⩽0.03–0.5	0.25	⩽0.03 – 0.5
MEPM	0.015	⩽0.008 – 0.5	⩽0.008	⩽0.008 – 0.5	0.015	⩽0.008 – 0.5
PAPM	⩽0.008	⩽0.008 – 0.12	⩽0.008	⩽0.008 – 0.12	⩽0.008	⩽0.008 – 0.03
TBPM	⩽0.008	⩽0.008 – 0.06	⩽0.008	⩽0.008 – 0.06	⩽0.008	⩽0.008 – 0.06
EM	>4	2 – >4	2	⩽0.12 – >4	>4	⩽0.12 – >4
CLDM	>4	⩽0.12 – >4	⩽0.12	⩽0.12 – >4	>4	⩽0.12 – >4
VCM	0.25	0.25 – 0.5	⩽0.12	⩽0.12 – 0.25	0.25	⩽0.12 – 0.25
TFLX	⩽0.12	⩽0.12 – 0.25	0.25	⩽0.12 – 0.5	⩽0.12	⩽0.12 – 0.25

PCV13, 13-valent pneumococcal conjugate vaccine; MIC, minimal inhibitory concentration; PCG, penicillin G; ABPC, ampicillin; CTX, cefotaxime; CDTR, cefditoren; CTRX, ceftriaxone; MEPM, meropenem; PAPM, panipenem; TBPM, tebipenem; EM, erythromycin; CLDM, clindamycin; VCM, vancomycin; TFLX, tosufloxacin.

### Serotype distribution and antimicrobial susceptibility of *H. influenzae*

Serotyping and antimicrobial susceptibility were performed in 126 sputum samples in which *H. influenzae* was the dominant isolate. With serotypes, 125 strains were non-typeable, one strain was type f but type b strain was not detected. Antimicrobial susceptibility did not also change after PCV13 introduction. Of the 126 isolates, 92 (73.0%) were ABPC resistant (ABPC ⩾ 4) (Table 7). Among the isolates, 33 (26.2%) were *β*-lactamase-producing strains. *β*-lactamase non-ABPC-resistant strains had less susceptibility to third generation cephalosporin.

**Table 7.** Antimicrobial susceptibility of *Haemophilus influenzae* isolates from children with community-acquired pneumonia

*H. influenzae*	2016		2017		2018	
Number of strains	33		52		41	
	MIC_50_	MIC range	MIC_50_	MIC range	MIC_50_	MIC-range
PCG	8	0.12 – >8	8	0.12 – >8	>8	0.25 – >8
ABPC	4	0.25 – >16	8	0.25 – >16	8	0.5 – >16
CTX	0.5	⩽0.03 – >4	0.5	⩽0.03 – 4	0.5	⩽0.03 – 4
CDTR	0.12	⩽0.03 – 0.25	0.25	⩽0.03 – 0.5	0.12	⩽0.03 – 0.5
CTRX	0.12	⩽0.03 – 0.25	0.12	⩽0.03 – 0.5	0.12	⩽0.03–0.5
MEPM	0.25	0.03 – 1	0.25	0.03 – 1	0.12	0.03 – 0.5
PAPM	1	0.25 – >2	2	0.12 – >2	1	0.25 – >2
TBPM	0.5	0.06 – 2	0.25	0.06 – 2	0.25	0.06 – 1
EM	4	2 – >4	4	1 – >4	4	2 – >4
CLDM	>4	4 – >4	>4	2 – >4	>4	4 – >4
VCM	>2	>2	>2	>2	>2	1 – >2
TFLX	⩽0.12	⩽0.12 – 2	⩽0.12	⩽0.12 – 2	⩽0.12	⩽0.12 – 2

PCV13, 13-valent pneumococcal conjugate vaccine; MIC, minimal inhibitory concentration; PCG, penicillin G; ABPC, ampicillin; CTX, cefotaxime; CDTR, cefditoren; CTRX, ceftriaxone; MEPM, meropenem; PAPM, panipenem; TBPM, tebipenem; EM, erythromycin; CLDM, clindamycin; VCM, vancomycin; TFLX, tosufloxacin

## Discussion

This is the first study to directly prove the impact of PCV13 against hospitalised paediatric CAP following an observational surveillance study in Asia. Compared to before PCV13 introduction, hospitalised paediatric CAP incidence decreased significantly. Furthermore, the proportion of CAP due to *S. pneumoniae* decreased and the pneumococcal serotype changed from PCV13 serotypes to non-PCV13 serotypes. During the three study periods (2008–2018), health system and diagnostic methods of CAP and bacterial pneumonia were not changed in Chiba City. This phenomenon further provides evidence of the impact of PCV13. To date, reports on the effectiveness of PCV13 against paediatric CAP have been focused on children <5 years of age [4–8]. In 2012, after the introduction of PCV7, the incidence of CAP among those aged 5–15 years increased in our surveillance system (Fig. 2). The *Mycoplasma pneumoniae* epidemic and CAP due to other microbial pathogens influenced the overall incidence of CAP. A *M. pneumoniae* infection epidemic occurred in Japan from 2011 to 2012 [20]. One of the reasons why the incidence of CAP in older children increased in 2012 could be attributed to the epidemic of macrolide-resistant *M. pneumoniae* infection in Japan [20]. Our study revealed that after the introduction of PCV13, hospitalised CAP incidence decreased not only in children aged <5 years, but also in those aged 5–15 years. In the PCV13 period, almost all children aged ⩾5 years did not receive the full shots of PCV13 because it was introduced in Japan in 2013. According to a review paper, an indirect effect of PCV13 for both invasive pneumococcal disease (IPD) and pneumonia in adults has been observed [21]. Our results suggest that PCV13 also had an indirect impact on children aged 5–15 years.

Practically, among 232 hospitalised CAP children <5 years of age in this study, only six (6.6%) were PCV13 unvaccinated. Conversely, among 48 hospitalised CAP children 5–15 years of age, 27 (56.3%) were PCV13 unvaccinated. The rate of hospitalised CAP in unvaccinated children 5–15 years of age decreased from 10.3% (65/626) in 2008 to 9.6% (27/280) in 2018; however, not statistically different.

Diagnosis of bacterial pneumonia was an important issue in this study. The prevalence of invasive PP in children in developed countries is low [22]. There were only seven children with CAP complicated by bacteraemia among the 1399 CAP patients in this study. To estimate the direct effect of PCV, several diagnostic methods have been used, including serological diagnosis, pneumococcal-specific PCR and urinary antigen [23–25]. In our surveillance, we utilised sputum sample for the diagnosis of bacterial pneumonia because sputum can be obtained easily and non-invasively. Both the Japanese and the US guidelines recommend sputum Gram staining and culture for the pathogenic diagnosis of paediatric CAP [16, 26]. The diagnostic yields of sputum culture are limited by potential contamination from the upper respiratory tract. We combined a washing technique with a semi-quantitative culture approach to evaluate pathogenic bacteria in our previous studies [11, 12]. Given that *S. pneumoniae* is rarely isolated from blood culture in children with pneumonia, the washed sputum method used in this study was reliable and can be used to identify the pathogen in children. This enabled us to investigate the impact of PCV13 on the incidence of non-invasive PP.

Washed sputum culture has also provided information on how PCV13 protects against pathogenic bacteria other than *S. pneumoniae*. In our study, after PCV13 introduction, PP incidence decreased; however, the number of pneumonia cases caused by *H. influenzae* and *M. catarrhalis* remained unchanged.

In terms of the serotype replacement of *S. pneumoniae*, after the PCV13 introduction, the causative *S. pneumoniae* strains changed from PCV13 type to non-PCV13 type in our study. Olarte *et al*. [27] reported that PP requiring hospitalisation significantly decreased in children after PCV13 introduction in the USA. The most common serotypes were 3, 19A and 35B in 2014, 4 years after PCV13 introduction. In our surveillance data, before PCV introduction, most of the isolated *S. pneumoniae* strains were 6B, followed by 19F and 23F. After PCV7 introduction, the isolated strains changed from PCV7 serotypes to non-PCV7 serotypes, mainly 6C, 15A, 15C and 19A. In this study, after PCV13 introduction, no PCV7 serotypes were detected and the incidence of PCV13 serotypes among isolated strains gradually decreased, 4 years after PCV13 introduction in Japan. The non-PCV13 serotypes isolated from participants during the study period varied. Of the non-PCV13 serotypes, serotypes 35B, 11A and 15A were the most frequently identified isolates; these serotypes and also 24B, were not isolated in 2008 and 2012 [11, 12]. In particular, serotype 35B has increased since 2017.

The phenomenon of serotype replacement has been reported for other respiratory infection and colonisation in Japanese children. Nakano *et al*. [28] recently reported the serotype distribution of *S. pneumoniae* isolated from paediatric patient with non-invasive diseases, the majority of which were otitis media during 2012–2014. According to their results, serotype 19A decreased and serotype 15A and 35B increased during the study period [28]. Ubukata *et al*. [29] also reported changes in serotypes of pneumococcal isolates collected between pre-PCV7, PCV7 and PCV13 periods in children. According to their report, among children, the proportion of PCV7 serotypes decreased rapidly from 73.3% during the PCV7 period to 2.3% during the PCV13 period. In particular, the number and proportion of nine serotypes (10A, 12F, 15A, 15B, 15C, 22F, 24F, 33F and 35B) increased significantly after the introduction of PCV7 and PCV13. Thus, even though the overall *S. pneumoniae* and CAP hospitalisation incidence has decreased, monitoring serotype replacement is important.

Concerning antimicrobial susceptibility, recovery of PCG susceptibility was reported after PCV7 introduction, because many PCV7 serotypes had penicillin-resistant strains [29]. In our study, also, after PCV7 introduction, antimicrobial susceptibility increased and the low resistant rate persisted after PCV13 introduction. However, several non-PCV13 serotype PCG-resistant strains existed, including MDR PMEN clone strains especially 15A (ST63). Serotype 35B (ST 558), which is a single locus variant of PMEN clone Utah^35B^-24 (ST377), was also a major non-PCV13 strain with reduced susceptibility to PCG in our study. These strains also showed reduced susceptibility to cephem and carbapenem. Another study on the antimicrobial susceptibility of *S. pneumoniae* clinical isolates, conducted among children in Japan, showed the spread of *S. pneumoniae* with a reduced susceptibility to PCG in non-vaccine serotypes 15A (ST63); 23A (ST338, 5242); 6C (ST242, 5832); and 35B (ST558) [30]. Among strains isolated from otitis media samples, the PCG-resistant serotypes 15A and 35B have also increased. Serotypes 15A, 3 and 35B most often belong to STs 63, 180 and 558 [31]. In other countries, the increase in MDR serotype 35B is also directly related to the expansion of ST558 clonal complex and the emergence of vaccine escape recombinant 35B (ST156) due to capsular switching [32]. Serotype 15A is also known to have one of the MDR pneumococcal serotypes [33]. Almost all MDR 15A isolates were ST63 variants, whereas susceptible 15A isolates were clonally diverse. The rise in serotype 15A suggests that PCVs will need ongoing adaptation [34]. Therefore, there is need to focus attention on these types of strains. In Japan, the rate of macrolide-resistant of *S. pneumoniae* is higher than in other countries [29]. In our study, all isolated strains in 2016 were macrolide resistant. However, several macrolide-sensitive strains have been isolated since 2017.

Non-typeable *H. influenzae* is also an important bacterial pathogen that causes CAP in children [35]. In our study, *H. influenzae* was the most prevalent bacterial pathogen isolated from sputum in study participants. Serotype distribution of *H. influenzae* is quite different from that of *S. pneumoniae*. Almost all isolated strains are non-typeable. Only one serotype f strain was isolated from sputum culture. After the introduction of Hib conjugate vaccine in Japan, invasive Hib infection dramatically decreased [36]. Pneumonia caused by Hib has also been eliminated. In terms of the antimicrobial susceptibility of *H. influenzae,* ABPC resistant rate was 73.0% including the *β*-lactamase-producing rate of 26.2%. Both resistant rates are higher than the rate in other nationwide studies in children from Japan [37, 38]. *β*-lactamase ABPC resistant *H. influenzae* (BLNAR) has a major resistant pattern of ABPC resistance in Japan and BLNAR strains were also less susceptible to cephems. We have to monitor the tendency of ABPC resistant *H. influenzae* isolated from CAP in children continuously.

Our study has limitations. The diagnosis of pneumonia was not based on a standardised method; for example, the definition for radiological pneumonia was developed by a World Health Organisation (WHO) group [39]. The WHO criteria are not being utilised in Japan. Thus, we followed the same method used in our previous studies [11, 12].

In conclusion, since the introduction of PCV for children, the incidence of hospital admissions for paediatric CAP has decreased in Japan. Culture-proven PP due to PCV13 serotypes has dramatically decreased. This phenomenon suggests the direct impact of PCV13 against paediatric hospitalised CAP. Furthermore, after PCV13 introduction, the incidence of hospitalisations due to CAP decreased not only in children aged <5 years but also in children 5–15 years of age. Conversely, serotypes less susceptible to penicillin, namely, non-PCV13 serotype 15A (ST63) and 35B (ST 558), have become the main non-PCV13 serotypes in Japan. PCV13 is effective for the reduction of hospitalised CAP in children and it is important to maintain the high PCV13 vaccination coverage to control CAP in children. Continuous surveillance of paediatric CAP is necessary to detect emerging pathogens for appropriate management of pneumonia in children.
